# “It changes everything”: Understanding how people experience the impact of living with a lower-grade glioma

**DOI:** 10.1093/nop/npae006

**Published:** 2024-01-29

**Authors:** Ben Rimmer, Michelle Balla, Lizzie Dutton, Sophie Williams, Joanne Lewis, Pamela Gallagher, Tracy Finch, Richéal Burns, Vera Araújo-Soares, Fiona Menger, Linda Sharp, Ways Ahead Study Team, Sara Erridge, Pauline Sturdy, Catherine McBain

**Affiliations:** Population Health Sciences Institute, Newcastle University Centre for Cancer, Newcastle University, Newcastle upon Tyne, UK; Faculty of Medical Sciences, Newcastle University, Newcastle upon Tyne, UK; Population Health Sciences Institute, Newcastle University Centre for Cancer, Newcastle University, Newcastle upon Tyne, UK; Newcastle upon Tyne Hospitals NHS Foundation Trust, Newcastle upon Tyne, UK; Newcastle upon Tyne Hospitals NHS Foundation Trust, Newcastle upon Tyne, UK; School of Psychology, Dublin City University, Dublin, Ireland; Department of Nursing, Midwifery and Health, Northumbria University, Newcastle upon Tyne, UK; Faculty of Science, Atlantic Technological University, Sligo, Ireland; Health and Biomedical Strategic Research Centre, Atlantic Technological University, Sligo, Ireland; Population Health Sciences Institute, Newcastle University Centre for Cancer, Newcastle University, Newcastle upon Tyne, UK; Centre for Preventive Medicine and Digital Health, Department for Prevention, Medical Faculty Mannheim, Heidelberg University, Heidelberg, Germany; School of Education, Communication and Language Sciences, Newcastle University, Newcastle upon Tyne, UK; Population Health Sciences Institute, Newcastle University Centre for Cancer, Newcastle University, Newcastle upon Tyne, UK

**Keywords:** lower-grade glioma, qualitative, quality of life, supportive care needs

## Abstract

**Background:**

Quantitative studies show people living with a lower-grade glioma (LGG) often report low health-related quality of life. However, it is unclear how this impact is experienced; resulting supportive care needs are also poorly understood. We explored how people experience the impact of living long-term with an LGG, to help identify potential supportive care needs.

**Methods:**

We conducted semi-structured interviews with a diverse group of people with LGG (*n* = 28) across the United Kingdom, who had completed primary treatment (male *n* = 16, female *n* = 12, mean age 54.6 years, mean time since diagnosis 8.7 years). Interviews were transcribed and inductive thematic analysis was conducted.

**Results:**

Four themes relating to the impact experiences of people with LGG were generated: “*Emotional response to the diagnosis*,” “*Living with the ‘What ifs’*,” “*Changing relationships*,” and “*Faltering independence*.” These reflect participants’ experiences with symptoms (eg, fatigue, seizures) and impairments (eg, motor dysfunction, cognitive deficits), and how these, in turn, drive impacts on daily living (including on work, relationships, social activities, and transport). Participants spoke about their experiences with profound emotion throughout.

**Conclusions:**

People with LGG can experience wide-ranging everyday impacts and may have extensive supportive care needs. This study highlights how this impact is experienced and what it means to people with LGG. Best practice suggestions for conducting comprehensive needs assessments tailored to those with LGG, and the development of personalized plans to meet those needs, would be a critical step to ensure that people with LGG are best supported in living with their condition.

Gliomas are the most common malignant tumor of the brain.^1^ Approximately 15% of all gliomas are lower-grade gliomas (LGG). These are largely diagnosed at a critical time in working and family lives in adults in their 30s and 40s.^2^ They are mostly incurable and will likely progress to a high-grade glioma (HGG),^3^ limiting life expectancy to 5–15 years depending on the subtype.^2,4^ Hence, LGGs are distinct from other cancers in that people may live for long periods with a condition likely to be terminal.

People with LGG can experience wide-ranging, and often co-occurring, symptoms and impairments that are both more general cancer-related (eg, fatigue, pain) and quite tumor-specific (eg, seizures, cognitive deficits).^5,6^ Individuals also report considerable emotional and psychological impact; largely influenced by future uncertainty surrounding their prognosis.^7^ Indeed, there is a high prevalence of mental health disorders in people with glioma.^8^ Quantitative studies indicate that these symptoms and impairments adversely impact health-related quality of life (HRQoL) in people with LGG.^9,10^ HRQoL is markedly worse in people with LGG than people with meningiomas^11^ and non-cancer controls, and remains generally poor over time^5^; in particular, fatigue and the emotional impact may persist long-term post-diagnosis with an LGG.^12^

Social decline following a brain tumor diagnosis can follow a similar trajectory to physical decline.^13^ Cubis et al. outline the social trajectory of a brain tumor more generally, highlighting the loss of pre-illness networks (eg, work, peer)^14^ and how physical, cognitive, and psychological factors can present a barrier to social interaction.^15^ Though potentially applicable to people with LGG, these studies largely included people with HGGs, which are more aggressive,^1^ so participants may have experienced a more intensified impact over a shorter duration. People with LGG may encounter social challenges, such as strained relationships, financial, or work-related problems^6^; certainly return to work rates are typically low in people with LGG.^16^ However, patient-reported outcome measures may not sufficiently encompass these impacts.^17^

In terms of qualitative research, there is a paucity of data which may help us to understand how people experience the impact of living long-term with an LGG. One previous study focused on the experiences of onset and diagnosis among people with LGG.^18^ Edvardsson et al.^19^ highlight the broad range of illness-related problems perceived by people living with a grade 1 or 2 brain tumor; however grade 1 tumors are distinct from LGGs, as they have a more favorable prognosis.^3^ Further, they focused on identifying areas of impact, rather than how the impact was experienced.

It is crucial to consider the impact experiences of people with LGG to understand what it means to them; this is important to help recognize potential supportive care needs and develop appropriate supports. For example, seizure burden is commonly associated with worse HRQoL in people with LGG^5^; we need to understand how this impact is experienced (eg, inability to drive) and why it is important to people with LGG, to fully comprehend the extent of the impact (eg, unable to transport to work).

Therefore, this study aimed to explore how people experience the long-term impact of living with an LGG. This will expand on existing knowledge to understand what is important to people with LGG and how symptoms and impairments drive the impacts on daily living.

## Methods

### Design

This qualitative study, part of the multi-method Ways Ahead project,^20^ was descriptive in design and sought to recognize the diverse and subjective experiences of participants in an area where little is known.^21^ We used semi-structured interviews to generate data on the experiences of people with LGG; the data analyzed here focused on how people experience the impact of living with an LGG. We have reported elsewhere from this dataset on the strategies used by people with LGG to self-manage this impact^22^; the 2 papers are thus complementary. Ways Ahead was reviewed and approved by the Wales Research Ethics Committee (REC ref: 20/WA/0118).

### Participants and Recruitment

Individuals were eligible if they were aged ≥18 years at diagnosis, resided in the United Kingdom (UK), and had completed primary treatment, or were stable under observation, for a grade 2 astrocytoma, or a grade 2 or 3 oligodendroglioma.^23^ Participants were excluded if they were non-English speaking or their clinical team judged they had severe psychological or social problems and there was a risk that an interview would cause further distress.

Potentially eligible people with LGG were identified through collaborating National Health Service (NHS) sites and the Brain Tumour Charity’s networks. Purposive sampling was used to ensure we recruited a range of ages, sex, diagnoses, and time since diagnosis (<5 years, ≥5–10, >10 years).

For NHS sites, people with LGG were identified from medical records and given an information sheet by a healthcare professional. For the Brain Tumour Charity networks, BR disseminated a flyer advertising the study, with the information sheet attached. The researchers conducting the interviews were briefly introduced in the information sheet. People with LGG were asked to call or email the study team to register their interest. BR and LD subsequently called each interested person to confirm eligibility, afford the opportunity to ask questions, and, if the individual was confirmed as eligible and wanted to proceed, arrange a convenient interview date and time. Participants were recruited August 2020–May 2022.

### Data Generation

One-to-one interviews were conducted by BR (Male, MSc, Research Assistant) and LD (Female, PhD, Research Associate): both trained and experienced in qualitative research. All interviews were conducted remotely, via a phone or video call (eg, Zoom or Teams), as per interviewee preference. To support participants who may have had cognitive impairments, such as memory and processing speed limitations, we offered an interview topic overview in advance and, in the interview, allowed ample time to consider and respond to each question.

Audio-recorded consent was acquired immediately prior to each interview. We also collected basic demographics (including sex, age, employment and relationship status, years of education, number of dependents) and clinical and tumor-related information (including diagnosis, date of diagnosis, tumor location and laterality, treatment, IDH1-mutation and 1p19q codeletion status). We asked participants recruited through the Brain Tumour Charity for their main treating hospital and consultant. For all participants, we asked their treating hospital to confirm clinical and tumor-related details; self-reported information is reported where confirmation could not be obtained.

Interviews were semi-structured following a topic guide (Supplementary Material), which was informed by the literature and expert knowledge, and modified following review by a brain tumor Patient and Public Involvement (PPI) panel and discussions with healthcare professionals (JL and SW). Throughout data generation, the topic guide was used flexibly; topic order varied for each interview, depending on what the participant chose to speak about. Any new issues raised were added and explored in subsequent interviews.

To begin, participants were asked to broadly reflect on life following diagnosis. Participants’ experiences of what areas of their life and functioning were affected by the tumor and its treatment (eg, cognitive, physical, psychological) and the impacts on aspects of daily life (eg, work, transport, relationships, finances) were then explored. For each area, participants were asked how they were impacted, what type of support was needed and in which situations, and whether support was received. Probe questions were used to explore further, and participants could raise any additional issues of importance to them. Finally, participants were offered a £20 voucher to thank them for their time, and a post-interview sheet with details of charities and helplines, should they experience distress post-interview. Interviews were audio-recorded and lasted on average 102 min (range 54–167 min). Field notes were taken by the researchers during each interview for their own reference.

### Data Analysis

Interviews were transcribed verbatim, anonymized, and checked for accuracy against the audio-recordings. The present analysis used a flexible, data-driven approach, in line with inductive thematic analysis^24^; this allowed us to develop patterns of meaning from the impact experiences described by people living with an LGG.

Following familiarization with the data, 2 trained researchers (BR and MB) independently generated initial codes, using NVivo, for a sample of transcripts (*n* = 6 of 28); similarities and differences in preliminary codes were discussed between the researchers. BR coded the remaining transcripts, adding any new codes as needed; findings and any uncertainties were discussed with the study team as analysis progressed. Potential themes at the semantic level were then constructed from these codes and discussed with the study team. Data sufficiency was determined by the perception that we had generated sufficient data to support and understand the impact experiences of people living with an LGG.^25^ Final themes and subthemes were refined and defined by BR, and are reported below with illustrative quotes. Each participant was provided a summary of findings and given the opportunity to offer feedback.

## Results

### Participant Characteristics

Thirty-nine people with LGG registered interest; 4 were excluded for non-completion of primary treatment (*n* = 2), ineligible diagnosis (*n* = 1), and residence outside the UK (*n* = 1). Of 35 eligible people, 28 were interviewed (10 recruited through NHS sites; 18 through the Brain Tumour Charity). There were 16 male and 12 female participants; mean age at the interview was 50.4 years (median 52 years, range 22–69 years) (Table 1). Diagnoses comprised grade 2 oligodendroglioma (*n* = 10: IDH1-mutant, yes *n* = 7, no *n* = 2, unknown *n* = 1; 1p/19q codeletion, yes *n* = 9, unknown *n* = 1), grade 3 oligodendroglioma (*n* = 9: IDH1-mutant, yes *n* = 6, no *n* = 1, unknown *n* = 2; 1p/19q codeletion, yes *n* = 7, unknown *n* = 2), and grade 2 astrocytoma (*n* = 9: IDH1-mutant, yes *n* = 6, no *n* = 1, unknown *n* = 2; 1p/19q codeletion, no *n* = 7, unknown *n* = 2). Mean time since diagnosis was 8.7 years (range 1–18 years).

**Table 1.** Lower-Grade Glioma Participants’ Characteristics at Time of Interview

Characteristic	*n*	Characteristic	Mean (range)
Diagnosis^a^		Time since diagnosis (years)^a^	8.7 (1–18)
Grade 2 oligodendroglioma	10	Time since radiotherapy (years)^a,c^	6.9 (0.7–17.8)
Grade 3 oligodendroglioma	9	Time since chemotherapy (years)^a,c^	3.4 (0.1–13.5)
Grade 2 astrocytoma	9	Full-time education (years)	15.8 (11–20)
IDH-mutation status^a^		Sex	*n*
Yes	19	Female	12
No	4	Male	16
Unknown	5	Age	
1p/19q codeletion status^a,b^		≤40	4
Yes	16	41-50	8
No	7	51-60	11
Unknown	5	>60	5
Treatment^a^		Dependents	
Surgery	28	None	18
Radiotherapy	22	One	3
Chemotherapy	17	Two	6
Tumour location^a^		Three	1
Frontal	18	Employment status	
Temporal	3	Full-time employee	8
Parietal	3	Part-time employee	4
Overlapping regions	3	Retired	4
Unknown	1	Medically retired	6
Tumour laterality^a^		Unable to work	6
Right hemisphere	13	Relationship status	
Left hemisphere	15	Married	21
Dominant hemisphere	13	In a relationship	3
Non-dominant hemisphere	15	Single	2
		Widowed	2

^a^Clinical and tumor-related details were self-reported for 8 participants.

^b^All participants with 1p/19q codeletion were people with oligodendroglioma; all participants without 1p/19q codeletion were people with astrocytoma.

^c^Time since radiotherapy and chemotherapy were not available for 2 participants.

### Overview of Findings

**Figure 1.**
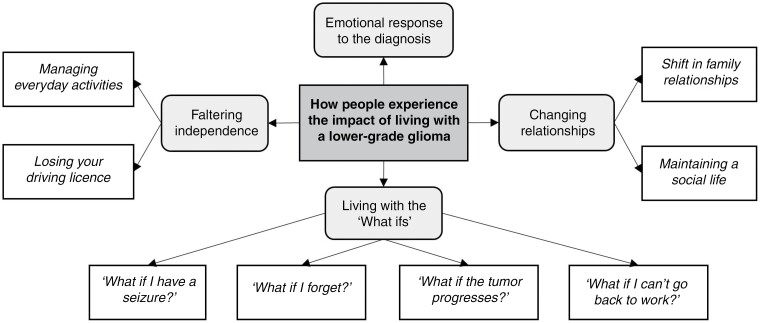
An overview of the themes and subthemes reflecting how people experience the impact of living with an LGG.

We constructed 4 themes to portray how people experience the impact of living with an LGG (Figure 1), namely: “*Emotional response to the diagnosis,”* “*Living with the ‘What ifs’*,” “*Changing relationships*,” and “*Faltering independence.*” These themes reflect participants’ experiences with tumor-related symptoms (eg, fatigue, seizures) and impairments (eg, motor dysfunction, cognitive deficits), and how these impacted on multiple areas of their daily lives. Participants spoke about their experiences with profound emotion throughout, as demonstrated in the illustrative quotes; additional supporting quotes are available in Table 2.

**Table 2.** Additional Supporting Quotes for all Themes and Subthemes, with Participant ID, Age, Sex, and Tumour Type at Interview

Theme	Subtheme	Illustrative quotes
Emotional response to the diagnosis		• “When I was first diagnosed, it was such a shock and there was a huge sense of not knowing what was going to happen and a loss of any feeling of control over my life.” (Pa29, aged 51, female, grade 3 oligodendroglioma) • “It does have a huge impact on me absolutely but I’m able to work, I’m able to have moments of joy and all of those kinds of things. So if I’m honest, I think I’m quite proud of how I’ve managed to deal with it.” (Pa40, aged 31, female, grade 2 astrocytoma) • “I think for the first couple of years I was in complete denial it [the tumour] was doing anything to my life, completely in denial.” (Pa19, aged 55, male, grade 3 oligodendroglioma) • “I really am lucky, I’ve seen other people with brain tumours who are far worse than I am as far as the impact it had on their life and the life of their loved ones and the disability that they’re having to cope with” (Pa15, aged 55, male, grade 2 astrocytoma)
Living with the “What ifs”1)2)3)	*“What if I have a seizure?”*	• “There’s been times when I’ve been in amongst a crowd of people and have a seizure. I’ve had somebody say, ‘Get off the bus.’ You know, I’ve had one where the driver said, ‘Get off the bus.’” (Pa25, aged 45, male, grade 2 oligodendroglioma) • “I wouldn’t venture as far as I normally would away from the village. And I think that was only because, you know, if you had a seizure or something if somebody would come across you or find you.” (Pa38, aged 55, female, grade 2 astrocytoma) • “I had a job at the time which involved getting a very early plane on the Wednesday morning . . . I started to worry about getting up that early and whether the seizures would come back. I found myself living a sort of lifestyle that I no longer wanted to be in because of the seizures.” (Pa35, aged 49, male, grade 2 astrocytoma)
	*“What if I forget?”*	• “Yesterday morning, I was four hours late [to take the medication], which on a twelve-hour cycle, I consider that to be really big. Now, I’m obviously catching up.” (Pa25, aged 45, male, grade 2 oligodendroglioma) • “There’s not much I can remember how to cook, now. I mean, I probably would be able to cook a sandwich. You know, cook the sausages. I’d be able to put stuff in the grill, but I wouldn’t remember how to do, like, a proper meal.” (Pa25, aged 45, male, grade 2 oligodendroglioma) • “I haven’t been able to walk around as much. [Before the diagnosis] I could always get back to my home. One day [wife] dropped me off at the Co-op. I said, ‘I’ll walk back up whilst you’re shopping.’ I did get lost so I had to put my Google App on my phone and work out where I was.” (Pa30, aged 61, male, grade 3 oligodendroglioma) • “I get reminders off the hospitals and doctors, which I am glad about, because I will forget. I always try and put things in my calendar in my phone and set an alert, but I don’t always remember to do it once I’ve got the information.” (Pa20, aged 47, female, grade 3 oligodendroglioma)
	*“What if the tumour progresses?”*	• “I have three kids. I think that’s a deep rooted parental thing that you feel like you’re letting people down if you’re on the way out . . . I let you down because you’re my kids, I’m meant to be here to look after you. I’m not meant to die.” (Pa28, aged 66, male, grade 2 astrocytoma) • “I feel like I’m in a nightmare and I’m never going to wake up . . . That’s part of the anxiety . . . it’s like you’re waiting for death, and it shouldn’t be that way. Or even worse, you’re waiting to be even more disabled than I am now.” (Pa9, aged 22, male, grade 2 astrocytoma) • “I get a little bit anxious about things, especially when I’m going for my scan, just until I get the results. So for a couple of weeks I’m on tenterhooks.” (Pa34, aged 66, female, grade 2 oligodendroglioma) • “Initially, you felt you were like a ticking time bomb, I would say, just waiting for something to happen. And if it did, you know, you were leaving your family behind” (Pa38, aged 55, female, grade 2 astrocytoma)
	*“What if I can’t go back to work?”*	• “I was made redundant because I was making mistakes. I’d forget something crucial to a film shoot. I just generally didn’t feel well . . . it’s just that fatigue, exhaustion, utter exhaustion.” - Pa18 (female, aged 55, grade 3 oligodendroglioma) • “To suddenly be told that you can’t do anything . . . I lost confidence after losing my job because someone saw it [the diagnosis] as a reason for me not to be working anymore, and it kind of kicks your confidence a bit.” (Pa20, aged 47, female, grade 3 oligodendroglioma) • “The financial side puts an awful lot of pressure. I mean my husband’s been working two jobs and we try and run a tight ship but the work, the hobbies, the driving, your interests, your social life, when you’re stripped of everything it’s very grounding.” (Pa18, aged 55, female, grade 3 oligodendroglioma) • “I’ve always enjoyed my work, I’m very lucky in that respect. In fact, getting back to work was a nice thing for me to do. I missed work when I was off. They’re long, lonely days especially when the weather’s crap and you can’t do much.” (Pa15, aged 55, male, grade 2 astrocytoma)
Changing relationships4)	*Shift in family relationships*	• “I was sitting at home thinking that I was useless . . . it’s always been a pretty 50/50 equal relationship and now I feel like I’m chief cook and bottle washer and he’s working still. I just feel that I don’t contribute as much as I did before I got the brain tumour.” (Pa29, aged 51, female, grade 3 oligodendroglioma) • “You’d have to ask my wife about my personality changes . . . She does say that I’m more short tempered and I am forthright and maybe not as ‘warm’ as I used to be.” (Pa31, aged 53, male, grade 2 oligodendroglioma) • “I can see that people worry about me. I could be sitting on the sofa reading a book or watching something on TV and when my mum’s here I catch her looking to see if I’m still breathing. Then she frets, ‘You’re doing too much, [Name].’” (Pa18, aged 55, female, grade 3 oligodendroglioma) • “It’s made us really, really value what we’ve got and really cherish that. It’s one of the good things in some respects. It has utterly cemented our relationship.” (Pa15, aged 55, male, grade 2 astrocytoma)
	*Maintaining a social life*	• “I wasn’t able to go out and visit friends or anything really because I just didn’t have the energy, just no energy at all.” (Pa35, aged 49, male, grade 2 astrocytoma) • “I constantly feel as though I’m on the outside, looking in. I’ve said that to the family, because I feel as though I have to invite myself to things.” (Pa20, aged 47, female, grade 3 oligodendroglioma) • “Some people, it’s [the diagnosis] just not something they’re willing or able to get on board with . . . maybe they’ll reappear when things are easier for a period of time, so they’ll be there for the fun times so to speak.” (Pa40, aged 31, female, grade 2 astrocytoma) • “My speech sometimes goes a bit slurry . . . I don’t think I would put my point across and join in the conversation as much as I used because of that.” (Pa38, aged 55, female, grade 2 astrocytoma)
Faltering independence	*Managing everyday activities*	• “I have to think what I’m ordering off the menu if we do go out for a meal because I feel embarrassed because I can’t cut up the meal because I can’t put pressure down with my right hand. I like steaks and whatever but I can’t cut them up.” (Pa22, aged 43, female, grade 2 astrocytoma) • “I’m going for the [job role] instead so I can catch some bad guys but it’s the fitness side I fail. I failed on the bleep test because I couldn’t turn around quick enough.” (Pa26, aged 37, female, grade 2 oligodendroglioma) • “I wouldn’t get on a bike. My balance isn’t . . . you know, there are certain things that would be dumb.” (Pa13, aged 52, male, grade 3 oligodendroglioma) • “If I washed my own pots, I was then too tired to go for a walk and get some fresh air and walk my dog.” (Pa17, aged 51, female, grade 3 oligodendroglioma)
	*Losing your driving license*	• “The only downside was I couldn’t drive, because once you have a seizure, you have to have a minimum year off. Being in sales, it’s not ideal.” (Pa11, aged 57, male, grade 2 oligodendroglioma) • “You feel like you’re, not a liability, but you feel like everybody has to almost give you something, that type of thing. I don’t want to be like that. I want to be the person who could give people lifts and that type of thing.” (Pa32, aged 46, female, grade 3 oligodendroglioma) • “The physical side, and losing my driving licence. It’s the independent side of me that gets the kick in the teeth.” (Pa26, aged 37, female, grade 2 oligodendroglioma) • “You don’t realize when you’ve driven all your life and I used to do crazy journeys and to have that taken away, that’s the biggest loss.” (Pa16, aged 69, male, grade 3 oligodendroglioma)

### Emotional Response to the Diagnosis

Most participants described feeling considerably emotionally impacted upon receiving their diagnosis; with initial shock, and ensuing feelings of anxiety, stress, low mood, and depression.

*“I’m a very, very positive person and with the first lot [of treatment], when I came around a bit and I was remembering things I wallowed for a while.”*

(Pa26, aged 37, female, grade 2 oligodendroglioma)

Many experienced difficulties with accepting the impact the diagnosis had, or would have in the future, on their life. Despite this, several participants felt “*proud*” with how they had coped with the challenges that followed the LGG diagnosis and its treatment. Many participants were conflicted by feeling emotionally impacted, while also feeling lucky compared to others; these feelings came from observing that their diagnosis was not as aggressive as a HGG, or that they had fewer symptoms than other people with LGG, and from recognizing the strength of their support network.

### Living With the “What ifs”

This theme encompassed how participants experience the impact of, and uncertainties related to, living with fatigue, cognitive impairment, seizures, and the incurable nature of an LGG diagnosis.

#### “What if I have a seizure?”.—

For several participants, the anxiety about having a seizure influenced their approach to daily life. Some avoided certain physical activities that they had enjoyed pre-diagnosis (eg, cycling, swimming), due to the anticipated negative repercussions of having a seizure while on the road or in the pool, for example. Others were unwilling to travel far from home due to possible negative reactions from other people if they had a seizure on public transport or worries about having a seizure in unfamiliar surroundings.

*“I am less willing to jog or cycle and worry about having a seizure when in the pool, because I think, ‘Well, if I did have a seizure while that was happening, I’d be in a much worse position.’”*

(Pa3, aged 45, male, grade 2 oligodendroglioma)

#### “What if I forget?”.—

For many participants, cognitive impairments frequently impacted numerous aspects of daily life, with potentially substantial consequences. They described possible security and safety repercussions of forgetting how to cook or leaving the front door open, for example. Several participants stressed how important and how challenging it was to remember their medication and health appointments. Some felt confined because they were losing familiarity with their local surroundings, often getting lost in places they once knew; this had implications for willingness to use public transport and travel alone.

*“Day-to-day, I’m leaving the house, I’ve got my bag, that’s all right, I can leave. I’ll leave, the door will be left open. And I’ll get out. And I’ll realise I haven’t got my keys. I haven’t got my phone. It’s a little bit of a joke sometimes because it’s so frequent.”*

(Pa17, aged 51, female, grade 3 oligodendroglioma)

#### “What if the tumour progresses?”.—

All participants spoke emotively about their worry surrounding future uncertainty due to the incurable nature of the diagnosis and potential for tumor progression; some feared they were ‘*a ticking time bomb’* or “*waiting to be even more disabled*.” Several expressed how anxiety worsened around scan appointments. Many found it challenging to remain positive: they found the uncertainty “*mentally draining*” and felt guilty about the prospect of “*leaving your family behind*” when they died. Participants described having to “*learn to live*” with how this uncertainty negatively impacted their ability to make decisions, both about smaller (eg, booking a holiday) and larger (eg, having children) aspects of life.

*“I was really afraid when in March, I booked a holiday for next August. I kept thinking, ‘Oh my God, will I get there?’ I have to have blind faith that I’ll be okay. So it changes everything. You have to learn to live with the changes, go with the flow.”*

(Pa18, aged 55, female, grade 3 oligodendroglioma)

#### “What if I can’t go back to work?”.—

This subtheme encompassed how people experience the uncertainties around returning to, and sustaining, work. Many participants highlighted the pressure of the financial consequences of employment changes (eg, going part-time). For some participants, work was a substantial source of social support, with time away from work resulting in feelings of loneliness. Those unable to work post-diagnosis (eg, due to fatigue and cognitive impairments) talked about how that “*kicks your confidence*,” with several feeling “*stripped*” of their identity, direction, purpose, and control over their life.

*“Because I wasn’t working and getting any positive feedback, I was sitting at home thinking that I was useless, pointless.”*

(Pa29, aged 51, female, grade 3 oligodendroglioma)

### Changing Relationships

This theme encompassed how participants experience the impact of living with an LGG on their relationships with partners and family, and their ability to engage in social activities with friends.

#### Shift in family relationships.—

All participants spoke emotively about how their diagnosis has influenced their relationships with family; largely acknowledging how emotional it had been for close family, particularly children. Some participants found new value in their relationships; though others recognized that family members were worrying about them more following their diagnosis. Some participants described no longer feeling equal in the relationship with their partner, because they could not contribute as much as they used to. Family relationships were further strained for several participants, by the impact of cognitive impairments (eg, forgetting plans) and personality changes (eg, short-temper).

*“I get on with things much as I used to really but emotionally, I’ve been flattened a bit by it all and I think that probably has had an impact on my family because I don’t think I’m a lot of fun sometimes.”*

(Pa28, aged 66, male, grade 2 astrocytoma)

#### Maintaining a social life.—

Most participants stressed the challenges with maintaining their social life and the consequent loneliness they felt. Many felt their relationships had changed with friends, attributing feelings of isolation mainly to a lack of willingness from others to “*get on board with*” the diagnosis and accept that things had changed. Fatigue had a substantial impact on several participants’ ability to go out with friends and engage in social activities. Those that mentioned attempts to socialize spoke about how they now lacked confidence due to difficulties with communication impairments, and sometimes felt overwhelmed by too many things going on around them in social situations.

*“I get this sort of brain flooding. If we have a lot of people over for a meal or something it gets a bit, there’s too much going on, the brain has taken too much.”*

(Pa5, aged 56, male, grade 2 oligodendroglioma)

### Faltering Independence

This theme encompassed how participants experience the impact of living with an LGG on their independence, particularly concerning their engagement with practical activities (eg, physical activity, transport).

#### Managing everyday activities.—

Several participants spoke emotively about how fatigue impacted their ability to do things for themselves in their daily life both generally, and specifically; the impacts were felt both on activities of daily living such as housework and on leisure activities like exercise. For some, completing one task or activity would leave them “*too tired*” to attempt another. For many participants, motor dysfunctions and issues with balance had wide-ranging consequences on their independence, from lacking the confidence to ride a bike, to feeling unable to cut up a meal. The combined effects of trying to deal with the side-effects of the tumor and its treatment while also managing everyday activities could feel overwhelming.

*“The rehabilitation can be soul destroying and you’re trying to get your life back and your personality back and everything is just so difficult and tiring.”*

(Pa36, aged 42, female, grade 2 astrocytoma)

#### Losing your driving license.—

The majority of participants reported losing their driving license following diagnosis and treatment; how long their license was revoked for was influenced by the presence of seizures. In those no longer able to drive, reactions were centered around the substantial impact this had on their independence, with some participants expressing that it was their “biggest loss.” This loss of independence had implications for participants’ work and hobbies. Some described feeling reluctant to be dependent on others (eg, take lifts because they could not drive), which limited their ability to engage with activities and interests outside of the home.

*“Because of the limitations of independence, relating to transport, I haven’t pursued hobbies or interests in the way that I have done maybe a year or two before surgery.”*

(Pa14, aged 66, male, grade 2 oligodendroglioma)

## Discussion

Quantitative studies show that people with LGG have low HRQoL and can face wide-ranging symptoms (eg, fatigue, seizures) and impairments (eg, poor motor dysfunction, cognitive deficits).^5^ To shed light on supportive care needs, our study aimed to explore how people experience the impact of living with an LGG. The four themes constructed from the data revealed how people with LGG experience wide-ranging impacts on activities of daily living, work, relationships, social and leisure activities, alongside emotional challenges in response to the diagnosis and the uncertainty of the prognosis.

Overall, our findings are coherent with the aspects of HRQoL evidenced to be impacted in people with LGG.^5,6^ The areas of impact reported were largely synonymous with the problems identified by Edvardsson et al. for grade 1 and 2 brain tumors^19^; an important addition from this study is that we stress the impact of seizures in people with LGG. In addition, by illuminating *how* people experience these impacts, our findings provide a more holistic insight, and suggest how different impacts might be interconnected. Moreover, our data relates to impact experiences after “active” (interventional) treatment; this means it complements Hayhurst et al.’s qualitative study of experiences of people with LGG being managed by the “wait and see” approach.^26^ Of note, the majority of our sample had received radiotherapy, which might have influenced the cognitive issues reported by participants.^27^

Our study participants reported considerable emotional and psychological burden following the initial shock of diagnosis, and ongoing fear of tumor progression and future uncertainty. Living long-term with this burden may be what contributes to persistent feelings of low mood^12^ and risk of mental health disorders,^8^ which have been reported in other studies. Our findings also underline the need for support with psychological adjustment in people with LGG.^28^

Some participants felt lucky to have a good support network, which is important because readily available emotional support can relieve the psychological burden.^13^ What the present study adds is how living with an LGG can hinder the maintenance of social connections both within the family and beyond. Participants particularly acknowledged the emotional burden their diagnosis had on their family, and spoke about how personality changes (eg, short-temper) and cognitive impairments strained their close relationships; this is similar to findings reported by people with HGG,^29^ though the data here shows these impacts are sustained longer-term in people with LGG. This is echoed in findings we have reported elsewhere from interviews with informal caregivers of people with LGG^30^; it also emphasizes that the support network of someone diagnosed with a LGG can have supportive care needs of their own.

Our findings are in line with the social trajectory of people with grade 1 to 4 brain tumors proposed in a qualitative metasynthesis.^14^ Specifically, participants in this study reported losing pre-illness support networks of colleagues and peers, with the decision or need to take time off work, or changes in employment contributing to feelings of loneliness, loss of direction and purpose. Losing their driving license could exacerbate isolation; communication impairments and fatigue further influenced participants’ confidence, and perceived ability, to engage in social activities. This supports findings on experiences of communication difficulties after glioma surgery.^31^ These examples of how functional impairments may drive the everyday impact of living with an LGG demonstrate the need to find ways to preserve social involvement and supportive relationships. As others have argued, this would likely help people to sustain their social identity and get the greatest benefit from their support network.^32,33^

An important challenge for participants was their ability to manage daily activities. Our findings are congruent with past work on the consequences of seizures in this population (eg, inability to drive)^5,34^; yet the present study goes further by highlighting how anxiety about the possibility of having a seizure also has ramifications, which further limit people’s lives. For example, such worries sometimes prompted the avoidance of certain physical activities (eg, swimming, cycling). Further, cognitive deficits, fatigue, and motor dysfunction presented difficulties with activities of daily living (eg, housework, using public transport). Overall, these impairments had implications for participants’ independence, leading to (further) changes in relationship dynamics with their partner and feelings of a loss of control over their lives. Hence, people with LGG may need several different types of support to manage individual impairments; for example, with seizures, there may be a primary need for these to be medically controlled, but also a need for psychological support to manage the unpredictability of, and anxiety about the consequences of having, potential seizures.

### Implications

**Summary of practical insights from this study**

- Need to develop best practice suggestions for needs assessments in clinical practice for people with LGG, capturing and contextualizing symptoms and impairments.
- Need to co-develop supportive care plans to meet identified needs for each individual.
- There is scope to develop self-management interventions for people with LGG, with adjustment- and problem-focused elements.

The box summarizes several practical insights from the study with implications for clinicians. The wide-ranging, and sometimes distinct, challenges and impacts faced by people with LGG demonstrate the importance of identifying what is important to each person and what support might be beneficial to them. There is a need to develop best practice suggestions for the conduct of needs assessments in clinical practice. Such needs assessments should be comprehensive in terms of the symptoms and concerns they capture and contextualize these by also assessing impact. For example, we showed here that fatigue can impact numerous aspects of life (eg, work, social activities, housework), hence, it is important to consider the context of the impact for the individual, so that support can be both tailored to the experience of having an LGG (as opposed to a different form of cancer) and personalized to the individual.^35^ Moreover, the assessment should be followed by working with the person with LGG to develop a supportive care plan for how best to meet identified needs. As part of that, it is important to acknowledge how people use support (eg, with assistance from family) and whether there are any barriers to engagement (access to support) that need to be overcome. Still, people with brain tumors may underestimate psychological, emotional, cognitive, and social changes^36^; thus, this needs to be dealt with sensitively, due to the risk of causing distress by getting people to realize that they are more impacted than they thought.

We have previously reported that people with LGG use many strategies to help them self-manage their illness.^22^ This indicates a willingness to engage in self-management, suggesting there is scope for more formal or structured self-management support to help people with LGG manage the consequences of their illness. Health professionals have a key role to play in encouraging, facilitating and supporting self-management.^37^ The present study outlines the areas of support that might be appropriate for the development of a self-management intervention targeted at people with LGG^20,38^; this could include adjustment-focused (eg, learning to live with future uncertainty) or problem-focused elements (eg, managing anxiety surrounding seizures; exploring strategies to compensate for cognitive impairments).^39^ This aligns with the goal of existing guidelines to provide the support that helps people to maintain independence and participate in valued activities.^40^ Still, future research should consider what may help or hinder an individual’s ability, capacity or willingness to engage with self-management (eg, strength of support network, executive functioning deficits hampering the ability to follow instructions), to ensure that the potential benefits of such support can be fully realized. Finally, routine monitoring of patient experiences of care and patient-reported outcomes would be of value to understand the impact of services and service changes (including needs assessments and supportive care plans), rehabilitation strategies, and self-management support, among people with LGG.

### Strengths and Limitations

Our study provides a novel understanding of the diverse and multi-faceted ways people experience the impact of living long-term with, and post-treatment for, an LGG. There was extensive data to support and understand the everyday impact of living with an LGG and the findings here are supported by multiple quotes; hence we are confident that reasonable data sufficiency was achieved. The relatively wide age range of participants means that people were likely at different stages of their lives; while this means that we captured diverse experiences, it also means we were unable to fully unpack how experiences might vary for people of different ages. For example, while issues around relationships, marriage, and children were evident within two themes, these may have been more strongly represented had participation been restricted to people in their 30s.

Due to corona virus disease 2019, all interviews were conducted remotely; however, this facilitated widespread recruitment across the UK,^41^ and may have helped people feel more comfortable to disclose sensitive information.^42^ It is possible that those recruited through the Brain Tumour Charity may have been self-selected, having more time, interest, and capacity to take part. Further, they may not represent those carrying on “as normal,” meaning the perspective of people who are managing well, with lesser impact, might have been missed. It is also possible that health professionals in collaborating sites may have been thoughtful in terms of who they approached to take part.

It has recently been noted that the exclusion of people with cognitive and communication impairments is not uncommon in LGG literature^5^ and that this has implications for the generalizability of findings. Although participants were given ample time in the interview to consider and respond to each question, we cannot discount the possibility people with more limited capacity were discouraged by the expected interview length (approx. 90 min) and chose not to take part. Future research might consider offering multiple, shorter interviews to mitigate the risk of fatigue and further support participation. Finally, participants did not undergo assessments of their abilities; such information (eg, in relation to cognition) may have been useful to set the findings in a clinical context.

## Conclusions

This study explored how people experience the impact of living long-term with an LGG. Our findings point to extensive supportive care needs, pertaining to psychological wellbeing, independence, and social identity among this population; these are driven largely by challenges with fatigue, seizures, cognitive deficits, and the emotional impact of living with an incurable condition. In the short-term, the findings will be of value to health professionals involved in the follow-up care of people with LGG. Longer-term, best practice suggestions for the conduct of comprehensive needs assessments tailored to those with LGG, and the development of personalized plans to meet those needs should be considered. This would be a critical step to ensure that people with LGG are best supported in living with their condition.

## Supplementary Material

npae006_suppl_Supplementary_Material
