# Supplementary material for: “It changes everything”: Understanding how people experience the impact of living with a lower-grade glioma
Source: Neurooncol Pract. 2024 Jan 29;11(3):255–65. doi: 10.1093/nop/npae006 (PMC11085834; doi:10.1093/nop/npae006)
Supplement: npae006_suppl_Supplementary_Material [file npae006_suppl_supplementary_material.pdf]

Ways Ahead

Improving support for people with brain tumours

Topic guide for patient interviews

The direction and content of the interview, the order in which topics are covered, and the precise wording of questions and probes, will be determined by the issues and topics raised by, and the personal circumstances and experiences of, the interviewee. This topic guide therefore functions as an issue checklist for the interviewer.

Introductory questions:

Would you like to start by telling me a bit about yourself?

And when were you diagnosed with a brain tumour?

Topics to cover:

- Experiences of living with a brain tumour*
  - Transition from treatment
  - Physical impact
  - Psychological impact
  - Cognitive impact
  - Emotions relating to brain tumour and its recurrence
  - Managing medications and health appointments
  - Relationships
  - Parenthood and caring roles
  - Work
  - Driving and other means of transport
  - Hobbies and interests
  - Finances
  - Seeking support
  - Healthcare support
  - Self-perception and societal roles
  - Coping and self-efficacy
- Most important aspect affected
- Desired support and intervention design preferences

**For each topic, cover the following:*

- What the impact was
- How it was managed
  - Challenges with managing impact
- What support was received
- What support was needed
- When it was most impacted
- When the support was needed

Closing questions:

Is there anything you would like to tell me that we haven't already discussed?
Do you have any questions for me?
